# Organellar protein multi-functionality and phenotypic plasticity in plants

**DOI:** 10.1098/rstb.2019.0182

**Published:** 2019-12-02

**Authors:** Sally A. Mackenzie, Hardik Kundariya

**Affiliations:** Departments of Biology and Plant Science, The Pennsylvania State University, 362 Frear North Building, University Park, PA 16802, USA

**Keywords:** WHIRLY, abiotic and biotic stress, epigenetics, retrograde signalling

## Abstract

With the increasing impact of climate instability on agricultural and ecological systems has come a heightened sense of urgency to understand plant adaptation mechanisms in more detail. Plant species have a remarkable ability to disperse their progeny to a wide range of environments, demonstrating extraordinary resiliency mechanisms that incorporate epigenetics and transgenerational stability. Surprisingly, some of the underlying versatility of plants to adapt to abiotic and biotic stress emerges from the neofunctionalization of organelles and organellar proteins. We describe evidence of possible plastid specialization and multi-functional organellar protein features that serve to enhance plant phenotypic plasticity. These features appear to rely on, for example, spatio-temporal regulation of plastid composition, and unusual interorganellar protein targeting and retrograde signalling features that facilitate multi-functionalization. Although we report in detail on three such specializations, involving MSH1, WHIRLY1 and CUE1 proteins in *Arabidopsis*, there is ample reason to believe that these represent only a fraction of what is yet to be discovered as we begin to elaborate cross-species diversity. Recent observations suggest that plant proteins previously defined in one context may soon be rediscovered in new roles and that much more detailed investigation of proteins that show subcellular multi-targeting may be warranted.

This article is part of the theme issue ‘Linking the mitochondrial genotype to phenotype: a complex endeavour’.

## 1. Plant adaptive features

As climate instability intensifies the challenges, both agricultural and ecological, to plant performance, increasing attention has been directed toward understanding natural mechanisms for plant resilience. Plants have diverse and active means of surviving abiotic and biotic stress. Much of their responsiveness is the consequence of rapid signalling and transport mechanisms [1] that integrate with response systems to evade [2], confront [3] or adjust to [4] the effects of stress. A plant's response to change in local environmental conditions can incorporate short-term memory, so that encountering a stress can leave the plant pre-primed for its recurrence later in the plant's life cycle [5]. Such ‘memory’ phenomena generally involve changes to local chromatin features that can facilitate more rapid gene response subsequently [6]. These genetic network and chromatin features represent important acclimation behaviours that allow the plant, as a sessile life form, to survive local, short-term environmental change.

Angiosperm evolution has produced remarkable species diversity due, in part, to the expansion of seed dispersal mechanisms, from pod shatter for 1 m distance to animal dispersal over kilometre distances [7]. Species invasion of new habitats requires mechanisms that accelerate evolution and adaptation; these can involve polyploidy [8,9], transposable element activity [10,11] and reproductive adaptations to cope with isolation [12]. Yet, these adjustments, which facilitate genomic plasticity, require a certain lag time. Consequently, more recent modelling of plant phenotypic plasticity in response to chronic changes in environmental conditions incorporates the role of epigenetics in the process.

Epigenetics is generally defined as heritable modifications in gene expression that do not involve genetic changes. Heritable epigenetics may refer to mother–daughter cell transmission or evidence of transgenerational effects [13]. Modelling an evolutionary scheme for accelerated and versatile resilience in plants, when contemplating ecological diversity, generally invokes some form of bet hedging in response to environmental fluctuation. A bet hedging model assumes that organisms have the capacity to suffer reduced fitness under ideal conditions in exchange for increased fitness under stressful conditions [14]. In plants, the most common examples include variation in seed germination rates, so that a smaller population number in the first year affords an opportunity for staggered multi-year germination [15]. Flower timing can also display variation to accommodate reproduction under unfavourable conditions that might improve over time [16]. Other, more subtle examples of bet hedging behaviours, and their molecular mechanisms, remain to be discovered.

## 2. Epigenetic control of phenotypic plasticity

A robust literature exists in support of epigenetic influence on plant environmental responses that may impact a population transgenerationally. A plant species in the forest understory experiences a diversity of light conditions. In the case of the herbaceous plant *Campanulastrum americanum*, individuals that experience excess light develop heritable high light tolerance, which serves to pre-adapt progeny and displays maternal transmission when crossed to corresponding shaded individuals [17,18]. Transgenerational effects of the environment on plant phenotype in natural populations can also affect drought response in species of *Trifolium* [19]. Not surprisingly, plants that reproduce asexually provide an excellent model for investigating epigenetic variation for environmental resilience in natural habitats. In apomictic dandelion, for example, epigenetic variation appears to contribute to heritable flowering divergence [20], ecological range expansion [21] and heritable drought stress [22].

The most well-studied epigenetically controlled trait in plants is flower time and, in particular, vernalization response. *Arabidopsis*, a particularly valuable model for studies of vernalization, relies on cold-induced epigenetic silencing of the floral repressor gene FLC during the vernalization process [23,24]. Studies of vernalization timing across natural accessions of *Arabidopsis* taken from a range of habitats show epigenetic variation to be important to this adaptive response [25].

There is evidence to suggest that other central plant gene networks may be targets for epigenomic effects as well. Circadian clock, jasmonate and ethylene response, and cold and light response are pathways that are regulated by *HISTONE DEACETYLASE 6 (HDA6)* [26], which participates in nucleosome compaction but also interacts directly with the cytosine methyltransferase *MET1* [27]. Transcription factor recruitment of chromatin-modifying components can provide targeting specificity for the epigenomic reprogramming of gene networks that are important to plant environmental responses [28–33]. For example, TOPLESS (TPL) binds and regulates the promoter of circadian clock genes CCA1 and LHY [34]. TPL also interacts with HDA6, serving to recruit histone deacetylation and differential methylation activity to alter the expression of these loci [35]. These types of interactions have significant implications for integrating gene network responses to environmental cues.

## 3. Organellar influences on plant adaptation behaviours

Plant mitochondria and plastids contain their own genetic information, distilled down following a series of post-endosymbiotic organelle-to-nucleus gene transfers that have left these genomes fairly rudimentary gene collections. The genes retained are thought to remain within the organelle to facilitate their redox regulation [36]. Organelles carry out their essential roles in energy transduction and environmental sensing but also conduct complex and multidimensional interorganellar communications within the cell [37]. Numerous excellent recent reviews have described the nature of what is termed retrograde regulation, comprised of organelle-originating signals that produce changes in nuclear response [38–40]. A number of organelle-derived molecules have been identified as signalling molecules in retrograde regulation, including reactive oxygen species and metabolites like β-cyclocitral [41], MEcPP (2-C-methyl-d-erythritol 2,4-cyclodiphosphate) [42,43], PAP (3′-phosphoadenosine 5′-phosphate) [44,45] and intermediates of the tetrapyrrole biosynthesis pathway [46]. More recently, it has become clear that multi-functional organellar proteins are also able to function within this retrograde signalling process. In many cases, these proteins may reside on an organellar inner or outer membrane in such a manner that a reactive oxygen species (ROS) or redox shift can trigger a change in conformation and transit of the protein to the nucleus [47].

Studies of post-endosymbiotic evolution provide valuable clues about plant adaptation to land. Polyplastidity, the presence of multiple dividing plastids per cell as opposed to a singular organelle, is uncommon among early algal lineages but was likely essential to land-plant adaptation [48]. The ability of particular plant lineages to adopt polyplastidity is thought to have influenced the magnitude of plastid-to-nucleus genetic transfers [49] as well as the subsequent specialization of plastid types [50]. The co-evolution of mitochondria and plastids as highly specialized cellular compartments has led to enhanced functional versatility of many nuclear-encoded organellar proteins by virtue of their dual targeting. Redirection of a protein to a new cellular location can be an important impetus to protein neofunctionalization [51]. Likewise, the spatio-temporal regulation of nuclear-encoded plastid proteins following promoter specialization [52] is an essential component of plastid multi-differentiation [53]. As a consequence, plastids are able to specialize for roles in photosynthesis, pigment biosynthesis, primary and secondary metabolic functions and stress signalling, to name a few. The distinct features of a plastid type are a function of their proteome composition. Because the majority of the proteome is nuclear-encoded [54], plastids can acquire distinct properties via spatio-temporal coordination of nuclear genes that encode these novel plastid functions [53].

Recent evidence indicates that plastids residing within the epidermal cell layer and in vascular parenchyma and bundle-sheath cells display features that are distinct from photosynthetic mesophyll chloroplasts. Epidermal and vascular plastids are 30% the size of a chloroplast and have a distinct proteome composition [55]. These small-sized organelles have been termed ‘sensory’ plastids due to their demonstrated enrichment for stress-associated proteins [53]. Separation of chloroplasts and sensory plastids, by GFP-assisted flow cytometry, reveal the chloroplast proteome to be enriched for photosynthesis and electron transport components, whereas sensory plastids contain a number of additional stress-related and vascular tissue-associated factors that function in stress response [53]. Perturbation of the sensory plastid can also result in epigenomic reprogramming and altered fitness phenotypes in the plant [53,56].

With regard to plant mitochondria, genome size and recombination activity are distinctively variable across vascular plants, ranging widely in size and configuration even among members within a genus [57]. This property contrasts with animal mitochondrial genomes, which are generally much more conservative in size, structure and gene composition. This seeming disparity between plant and animal evolutionary lineages is not well understood, but plant mitochondrial DNA recombination can produce dominant mutations that influence the reproductive biology of the plant. Maternally inherited mitochondrial mutations can induce male sterility, contributing to a natural reproductive system of gynodioecy [58]. This system involves plant transitions between hermaphrodite, with a capacity for self-pollination, and female, requiring a pollen donor for reproduction. When exploited by crop breeders, the male sterility component of a gynodioecious system constitutes an economical option for hybrid seed production [59]. Examples of this cytoplasmic male sterility (CMS) have been described in over 80 plant species [60], generally associated with the expression of mitochondrial gene chimaeras that arise from aberrant intragenic recombination [61].

CMS mutations generally encode proteins with hydrophobic domains and homology to components of the energy transduction pathway [61]. Expression of the mitochondrial sterility factor can be countermanded by nuclear genes known as fertility restorers. However, induction and suppression of the male sterility trait can also be controlled by regulating mitochondrial genome recombination, so that a subgenomic DNA molecule that encodes the sterility factor can be adjusted in relative copy number to influence its expression. Substoichiometric forms of the mitochondrial sterility gene are retained in the mitochondrial genome without influencing plant phenotype until amplified in copy number, leading to their detectable expression [62–64]. In an agricultural context, it is feasible to manipulate the CMS trait, together with fertility restorer genes, to permit the production of hybrid seed without manual emasculations. The system has proven to be a significant economic advantage for a variety of crops including rapeseed, onion, carrot, beet, sorghum, corn and rice [65].

In a natural ecosystem, the process is more complex. Plants within a population benefit from genetic diversity accrued through intercrossing, although plants finding themselves in isolation must rely on self-pollination to propagate [66]. A facultative system of gynodioecy interchanges both reproductive modes in response to environmental cues, with reproductive trait plasticity to facilitate reversibility within the system [66–68]. Sporadic incidence of CMS can arise in a population due to changes in mitochondrial recombination behaviour that lead to amplification of a quiescent CMS mutation, or by mutation of a nuclear restorer gene [58]. Male sterile individuals in the population facilitate population intercrossing and genetic diversity.

Nuclear fertility restorer genes most often comprise members of the expansive gene family of pentatricopeptide repeat (PPR) proteins [61]. This gene family has undergone remarkable expansion in size during plant evolution, often numbering in the hundreds of genes, with the vast majority participating in the regulation of organellar gene functions [69]. It appears reasonable to assume that the incidence of mitochondrial male sterility mutations, of selective advantage to the population, has been accompanied by concomitant selection for nuclear suppressors that may have served to diversify the PPR gene family [69]. This sterility gene-restorer gene dynamic bears resemblance to the coevolution of pathogenicity and plant disease resistance systems, which are similarly characterized by host gene family expansion as a means of diversifying defence [70].

The male sterile plant that finds itself isolated, however, will undergo flowering but must await successful pollination by a compatible pollen source. Recurrent, unsuccessful flowering in the absence of a pollination signal can lead to the incidence of spontaneous reversion to fertility, generally late in the life cycle [64,71,72]. In this situation, the mitochondrial genome again shifts its recombination behaviour to suppress the copy number of the male sterility-associated DNA molecule, thus recovering pollen fertility and successful, albeit low-frequency, self-pollination to the plant. This on/off mitochondrial switch for male sterility, and its environmental responsiveness, is an intriguingly overt example of phenotypic plasticity impacting fitness.

## 4. The protein MSH1 as a component of organellar stress signalling and plasticity

Detailed studies of the sensory plastid in angiosperms reveal organellar proteins with a unique influence on stress signalling. One such protein is MUTS HOMOLOG 1 (MSH1), a homologue to the mismatch repair and recombination factor *MutS*. MSH1 is nuclear-encoded, dual-targeted to mitochondria and plastids [63] and may be universally present in plants based on surveys to date of available algal, moss, fern and angiosperm species [73,74]; Y Wamboldt & SA Mackenzie 2016, unpublished). Characterized by six domains [73], the MSH1 protein encodes a DNA-binding motif (Domain I), an ATPase domain (V) and a GIY-YIG homing endonuclease domain (VI) [73], features implicating the protein in DNA binding and suppression of illegitimate recombination. These attributes presumably derive from its evolutionary origin.

MSH1 protein is expressed in epidermal, vascular parenchyma, meristem and reproductive tissues, and the gene is responsive to environmental stress. Under conditions of heat, cold, drought and excess light, *MSH1* steady-state transcript levels are markedly suppressed [55,75–77], while conditions of plant growth on sucrose result in a sharp increase in MSH1 transcripts ([78]; J Yang, N Zhao and SA Mackenzie 2019 unpublished). This sugar effect appears specific to sucrose and is not observed with other sugar sources. The spatio-temporal and environmentally responsive features of MSH1 are controlled by the gene's promoter [55].

Hemi-complementation experiments contrast phenotypic outcomes for the *msh1* mutant by complementing with mitochondrially targeted versus plastid-targeted forms of the MSH1 protein [79]. Thus, it is feasible to separate phenotype implications of the gene's mitochondrial versus plastid functions. Mitochondrial depletion of MSH1 enhances mitochondrial DNA recombination, producing asymmetric DNA exchange at unusually short intervals of sequence homology [80]. This change in recombination behaviour drives substoichiometric shifting in relative copy number of subgenomic DNA molecules [80,81] and a male sterility phenotype can emerge [64,82]. Thus, *MSH1* suppression following a change in environmental conditions can produce low-frequency transition to CMS. Enhanced *MSH1* expression in response to increased sucrose availability [78] implies that *MSH1* also responds to pollination cues. A sucrose increase signals successful pollination and may prove to be a regulator of fertility reversion. During flowering, a CMS plant in isolation receives no pollination cue, which triggers *MSH1* suppression and spontaneous reversion to fertility (J Yang, N Zhao and SA Mackenzie 2019, unpublished).

In the plastid, depletion of MSH1 signals a systemic stress state in the plant, and *msh1* mutant or RNAi suppression lines undergo dramatic changes in the expression of abiotic and biotic stress, circadian clock, phytohormone response and spliceosome pathways [83]. The *msh1* mutant also displays a range in altered phenotype intensity, with delayed maturation and flowering, altered leaf morphology, daylength sensitivity [79] and enhanced tolerance to drought [55], heat [75], cold [77] and excess light [76]. These effects are accompanied by genome-wide cytosine methylation changes that reflect epigenomic reprogramming [56].

**Figure 1.**
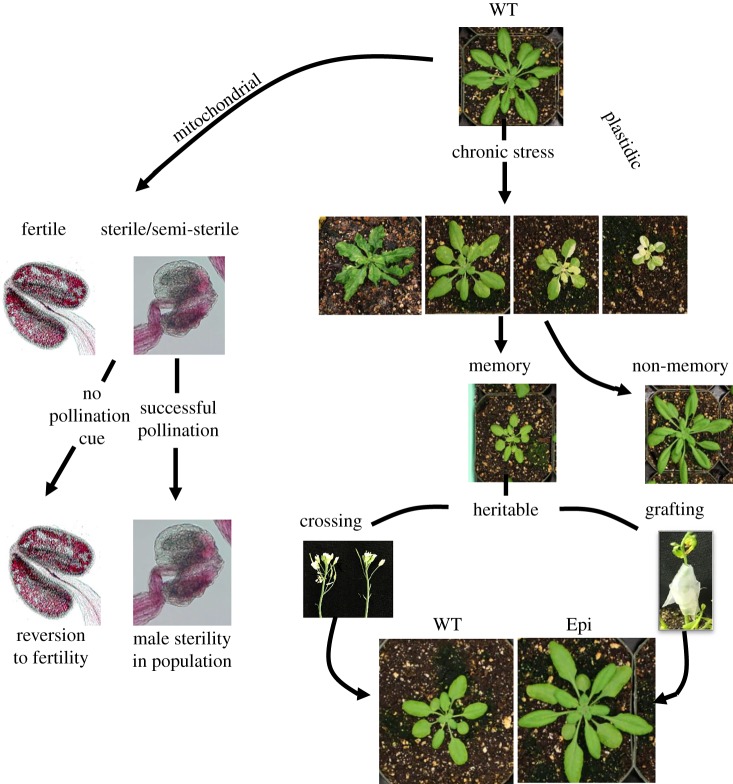
The MSH1 effect outlined in *Arabidopsis*. Plastidic and mitochondrial outcomes following MSH1 suppression and selection. Enhanced illegitimate recombination within the mitochondrial genome leads to substoichiometric shifting and expression of a male sterility trait. Lack of pollination in a CMS line can lead to low-frequency reversion to fertility, similarly involving MSH1 effects and mitochondrial subgenomic shifting. Depletion of MSH1 from the sensory plastid produces a variable stress-response phenotype that conditions transgenerational memory and enhanced stress tolerance. Memory line crossing or grafting with wild-type (WT) produces enhanced fitness traits (Epi). These effects are recapitulated across plant species, suggesting a role for MSH1 in plant adaptation.

To analyse *msh1* behaviour in more detail, it is feasible to create RNAi suppression lines that induce the *msh1* effect but segregate away the RNAi transgene in the subsequent generation. Resulting progeny segregates for the presence or absence of the transgene, which provides a test for epigenetic memory following *MSH1* reprogramming. Approximately 20% of the transgene-null progeny gives rise to an altered ‘memory’ phenotype more uniform than the original *msh1* parent phenotype, showing delays in maturity and flowering, reduced plant growth, altered circadian clock behaviour and a pale green leaf phenotype (figure 1; [56,79]). These ‘memory’ plants produce progeny that show 100% penetrance of the phenotype transgenerationally, a phenomenon that is recapitulated in *Arabidopsis*, soya bean, tomato, sorghum and tobacco [79].

## 5. MSH1 suppression leads to heritable epigenomic reprogramming in plants

The *msh1* memory state is transgenerationally stable and demonstrates genome-wide cytosine methylation repatterning. Because segregation of the MSH1-RNAi transgene produces both memory and ‘non-memory’ plants (transgene-null plants with no visible memory phenotype), it is feasible to compare wild-type, memory and non-memory types to discriminate epigenomic memory features. Surprisingly, gene-associated cytosine methylation levels are similar between the memory and non-memory full-sibs; what distinguishes the memory state is substantial methylation repatterning with particular reference to four gene networks: circadian clock, auxin response, phytohormone signal transduction and RNA spliceosome functions [84]. These observations are significant because memory and non-memory individuals derive from a single parent, so methylome changes that distinguish the two types are presumed to be associated with the memory phenotype. These observations are also crucial because they indicate that the methylome effects arising with stress memory are changes that appear to create a novel epigenomic state [84]. This finding contrasts with DNA methylation changes during gene silencing, which generally involve high-density, high-magnitude hypermethylation [85,86].

To understand how *msh1* memory might influence natural plant fitness, genetic crossing and grafting experiments have been carried out in various plant species. Reciprocal crossing of *msh1* memory lines with isogenic wild-type produces F2 populations with markedly enhanced variation in growth vigour, resilience and seed yield. Similar patterns of growth vigour have been observed in *Arabidopsis* [56,79], tomato [87], soya bean [88] and sorghum [89]. The enhanced vigour responds to selection over generations and diminishes to wild-type levels by the F6 generation. Such reversibility may be a key characteristic of epigenetic effects [90].

Grafting experiments that use *msh1* as rootstock with an isogenic wild-type scion also produce progeny that displays enhanced growth vigour, plant size and yield. Again, this augmented growth phenotype is differentially heritable for at least four generations, eventually diminishing back to wild-type levels. This epigenomic behaviour has been documented in soya bean [88], tomato [87] and *Arabidopsis* [56] and further confirmed by large-scale testing. The underlying basis for the robust growth outcomes, and any possible relationship to classic heterosis, remains to be uncovered.

## 6. Epigenomic reprogramming for *msh1* transgenerational memory, a component of ecological diversified bet hedging?

Figure 1 shows the MSH1-mediated process of epigenetic reprogramming to an enhanced fitness outcome. The *MSH1* gene may be widespread in plant lineages, although male sterility and epigenetic memory effects have only been documented in angiosperms, both monocot and dicot. Its association with both reproductive and stress response behaviours in the plant implicates *MSH1* in environmental adaptation. Methylome and gene expression changes in the *msh1* mutant and its derived memory state appear to target pathways that are central to plant stress response: circadian rhythm, auxin-related pathways, phytohormone signal transduction, spliceosome functions. Similarly, MSH1 regulation confers the ability to elicit and suppress a male sterility phenotype in response to changes in local population features. If these laboratory-derived patterns reflect naturally occurring behaviours, they insinuate plant mechanisms capable of adjusting heritable environmental responsiveness independent of genetic diversity.

From detailed ecological studies has emerged a model of adaptation by diversified bet hedging, which compromises maximal fitness under ideal conditions to improve fitness under stressful conditions [14], perhaps incorporating epigenetic processes [91]. This concept of compromised fitness may be pertinent to MSH1 phenomena. *MSH1* suppression signals chronic stress to the plant through sensory plastid-mediated changes, triggering system-wide stress responses. This perturbation results in a spectrum of effect intensity among *msh1* individuals, with significant differences in magnitude and diversity of gene expression responses across individuals within a single population [83]. Surviving plants give rise to a proportion of their progeny displaying heritable memory. In a natural habitat, this memory phenomenon can presumably provide transgenerational stress tolerance as the population enlarges. Crosses between memory plants and unmodified individuals produce progeny with enhanced fitness features that would be expected to expand local population competitiveness.

Studies have shown that organellar effects contribute to phenotypic canalization and the control of metabolic stochasticity in *Arabidopsis* [92]. Whereas phenotypic stability is important to maintaining metabolic homeostasis, the ability to retain and control some level of stochasticity within the system at different metabolic levels may contribute to bet hedging-based phenotypic plasticity in plants. Moreover, it is possible that particular core gene networks are interlinked with organellar signalling to permit rapid, programmed phenotypic responsiveness [93].

## 7. Are there other examples?

### (a) WHIRLY1

As unusual as the *MSH1* system appears to be, its multi-functional properties are not unique. Other plant organellar proteins display multi-functional behaviour to participate in environmental responsiveness. Whirly proteins are single-strand DNA-binding proteins that localize to the plastid, in the case of WHY1 and WHY3, and to the mitochondrion for WHY2 [94]. These proteins share structural and DNA-binding features in common [95], and their disruption, with *why1why3* double mutations or *why2* overexpression, leads to organellar genome instability [95–97]. The proteins appear to be associated with organellar nucleoids and likely suppress aberrant DNA recombination.

WHY1 has also been associated with stress responses within the nucleus. Evidence suggests that the WHY1 protein translocates to the nucleus from the plastid [98]. The nuclear-localized form of the protein has been shown to bind and influence telomere homeostasis in *Arabidopsis* [99], and to participate in salicylic acid signalling [100]. WHY1 may function in transcriptional repression of the *KP1* Kinesin-like protein gene [101], thought to function within the mitochondrion, implying that plastid-directed effects influencing WHY1 may alter mitochondrial regulation. WHY1 has also been suggested to repress WRKY53 expression, which leads to delayed leaf senescence [102]. All of these events are thought to confer changes in the plant in response to stress and ROS effects [103].

An intriguing hypothesis for WHY1 action was developed for modelling its behaviour in the plant cell [47]. Cross-tolerance or the general increase in resistance to a range of stresses following exposure to one, often leads to the activation of signalling pathways that alter both abiotic and biotic stress responses [104]. Redox and phytohormone signalling, in association with accumulation of ROS [105–107], can trigger this complex response, and WHY1 may participate in the retrograde signalling process initiated by plastid redox perturbation.

**Figure 2.**
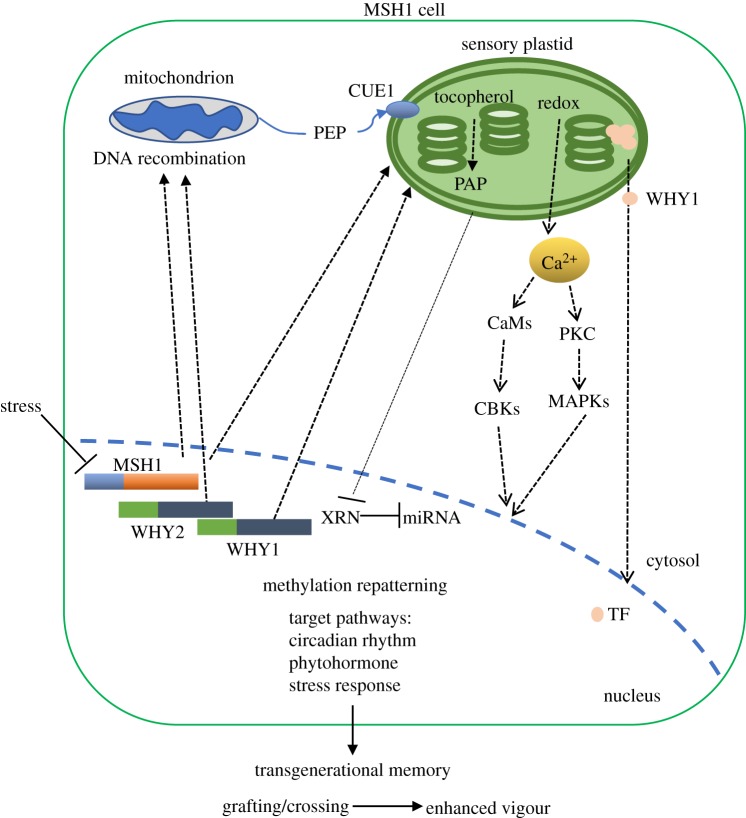
A simplified model of organellar influences on plant phenotypic plasticity. Stress-associated suppression of *MSH1* expression alters conditions within the sensory plastid of epidermal and vascular parenchyma cells [55]. These changes involve at least two retrograde signalling pathways to the nucleus, one including redox and calcium signalling [53] and the other tocopherol-mediated modulation of the PAP phosphonucleotide as a mediator of miRNA regulation [55,109]. Nuclear response to sensory plastid perturbation includes genome-wide cytosine methylation repatterning and altered expression of integrated stress response networks. Transgenerational memory induced by *MSH1* suppression gives rise, through crossing or grafting, to progeny with markedly enhanced growth vigour and resilience phenotypes [77,79,87,89]. WHY1 is localized to both mesophyll chloroplasts and sensory plastids [53], whereas WHY2 targets to mitochondria. However, WHY1 is distinctive in its apparent ability to transit back to the nucleus following plastid processing of the presequence [98]. This transiting allows WHY1 to function as a transcription factor in regulating stress responses. CUE1, a sensory plastid-specific PEP translocator, participates in tocopherol biosynthesis and the PAP-mediated retrograde signalling to regulate miRNA stability in the nucleus.

Similar to modelling of the *NONEXPRESSOR OF PR GENES 1 (NPR1*), which exists in oligomeric form within the cytosol but disperses to monomeric form for transit to the nucleus [108], WHY1 has been speculated to exist as an oligomer [95] at the interface of thylakoid membrane and nucleosome but as a monomer during nuclear transit [47]. Such a conformational change might be redox regulated. What makes this speculation particularly interesting is that both *MSH1* and *WHY1* appear to be involved in organellar DNA binding, thus nucleoid-associated, yet both appear to function beyond their DNA binding roles, with nuclear transiting by WHY1 and epigenomic reprogramming by MSH1. In both cases, it is likely that plastid perturbation, inciting changes in ROS levels and redox, condition these unusual stress signalling behaviours (figure 2).

### (b) CUE1

Plastids interconnect with mitochondria on multiple levels, often with the exchange of metabolic products [110]. For example, plastids import phosphoenolpyruvate (PEP), a glycolytic intermediate produced in mitochondria, as a precursor for at least four pathways, including the shikimate pathway [111]. Two plastid PEP transporter proteins are known in *Arabidopsis*, *PPT1* and *PPT2,* that localize to the plastid inner envelope membrane [112]. The PPT2 protein is found within mesophyll chloroplasts, whereas PPT1 is specifically expressed within the epidermal and vascular parenchyma cells and localizes to the sensory plastid [53,113]. Several observations suggest that the duplication and spatio-temporal regulation of PPT1 and PPT2 expression have resulted in PPT1 neofunctionalization.

*PPT1*, also known as *CAB UNDEREXPRESSED 1* (*CUE1*), has been confirmed to translocate PEP as a precursor to the shikimate pathway within the sensory plastid. The *cue1* mutant is characterized by defective expression of several light-regulated genes within the mesophyll but not in vascular tissue or epidermal cells, resulting in defective mesophyll chloroplast development [112,113]. These observations indicate that sensory plastid processes control mesophyll chloroplast development, and a *cue1* mutant displays a reticulated leaf phenotype of dark green venation with pale green lamina sections [114]. Double mutation of *cue1* with *eno1*, a plastid localized enolase that catalyses the glycolytic conversion of 2-phosphoglycerate to PEP, is lethal [115]. This observation implies that the mesophyll chloroplast-associated PPT2 is not able to compensate for *cue1* function and that the enolase and CUE1 functions may be adjoined within the sensory plastid.

Because PEP participates in the shikimate pathway, *cue1* can be complemented by exogenous application of aromatic amino acids [116]. This pathway is important to numerous stress processes as well as secondary cell wall biosynthesis [117]. Unexpectedly, this pathway also represents a potential link between sensory plastid processes and epigenetic functions in the plant. A *cue1* mutant acts as a second-site suppressor to mutants of *ROS1* [118], the demethylase (glycolase) that acts as a DNA methylation rheostat in the plant genome [119] and functions in genome-wide cytosine demethylation. The relationship of *CUE1* to genome methylation may be indirect and is not yet well understood.

*CUE1* also appears to participate directly in sensory plastid stress signalling. *CUE1*-mediated import of PEP is the first step in the biosynthesis of tyrosine, a precursor to tocopherols [117]. Tocopherol biosynthesis is essential for the accumulation of PAP. PAP is a component of plastid retrograde signalling and translocates from the plastid to the nucleus. Nuclear-localized PAP inhibits a class of exoribonucleases that degrade miRNAs so that under stress conditions, PAP signalling from the plastid serves to stabilize nuclear miRNAs that participate in stress response processes [109]. Likewise, PAP participates in ABA signalling to regulate stomatal closure [45] and drought response in the plant [44]. These observations point to an integrated sensory plastid–chloroplast–mitochondrial coordination that functions in metabolic, developmental and stress response processes of the plant (figure 2). The co-opting of metabolic linkages, gene duplications and protein dual targeting has supplied the system with a level of complexity that facilitates local and systemic stress responses that are vital to plant adaptation.

## 8. How did these systems evolve?

The evolutionary process of endosymbiosis was followed by the capacity of genes to transit, occasionally via RNA intermediates [120] or more often as entire genomic DNA fragments [121], to the nucleus. These transferred organellar sequences must undergo two subsequent processes, the acquisition of targeting presequences that provide the opportunity for protein transit back to the organelle and the procurement of a functional promoter to permit expression within the nuclear genome. It is these two functions that create intrigue regarding the evolutionary impetus for protein neofunctionalization. Studies have shown that the acquisition of an amino-terminal protein targeting presequence, and its expansion from singular to dual targeting, may involve mechanisms as seemingly incomplex as alternative translation initiation and leaky ribosome scanning [122–124]. Dual or mistargeting events serve to position proteins within new cellular environments, a stimulus for protein modification. Coupled with promoter evolution for spatio-temporal and environmental responsiveness, these factors appear to participate in MSH1, CUE1 and WHIRLY present-day multi-functionality. Interestingly, numerous proteins within the sensory plastid do not appear to be shared with the mesophyll chloroplast [53], implying that the sensory plastid may house additional components of environmental sensing yet to be characterized. Not surprisingly, numerous environmental responses have been mapped to plastids within the epidermis and vascular tissues [125–128]. The intensive research efforts now being directed toward abiotic and biotic stress effects in plants promise to reveal an even greater array of robust organelle-mediated defences in the future.

## Data accessibility

This article has no additional data.
